# Effects of Functional Red Pine Seed Direct-Drinking Oil on Constipation and Intestinal Barrier Function in Mice

**DOI:** 10.3390/antiox14010014

**Published:** 2024-12-25

**Authors:** Jie Li, Haonan Zheng, Jiahui Liu, Jie Ding, Qingqi Guo, Na Zhang

**Affiliations:** 1 College of Life Science, Northeast Forestry University, Harbin 150040, China; lijie@nefu.edu.cn (J.L.); jonathan@nefu.edu.cn (H.Z.); 2022120285@nefu.edu.cn (J.L.); dingjie@nefu.edu.cn (J.D.); 2 College of Food Engineering, Harbin University of Commerce, Harbin 150028, China

**Keywords:** red pine seed oil, fatty acids, direct-drinking oil, constipation, intestinal barrier, antioxidant

## Abstract

Constipation is a prevalent global health issue that greatly affects human well-being. However, many existing treatments are associated with side effects, necessitating the development of alternative approaches. In this study, a balanced fatty acid red pine seed direct-drinking oil (SFA:MUFA:PUFA = 1.14:1.08:1, n − 6:n − 3 = 4.17:1) was formulated using red pine seed oil as the base oil, blended with coconut oil, rice bran oil, and camellia oil. The study investigated the effects and mechanisms of this red pine seed direct-drinking oil in alleviating constipation in mice. Results showed that, compared to normal mice, constipated mice exhibited symptoms of dry stools, difficulty defecating, abnormal neurotransmitter levels, oxidative stress, and colonic tissue damage. Additionally, the protein expression levels of occludin and claudin-1 were reduced by 86.11% and 25.00%, respectively (*p* < 0.05), while mRNA expression levels decreased by 70.80% and 59.00% (*p* < 0.05). Red pine seed direct-drinking oil intake improved defecation, reduced serum levels of vasoactive intestinal peptide (VIP), endothelin-1 (ET-1), and nitric oxide (NO), and increased substance P (SP) levels. Furthermore, it also significantly elevated serum levels of superoxide dismutase (SOD) and catalase (CAT) (*p* < 0.05), alleviated colonic tissue damage, and upregulated the protein and mRNA expression levels of occludin and claudin-1 (*p* < 0.05). These findings suggest that red pine seed direct-drinking oil alleviates constipation in mice by enhancing intestinal motility, regulating serum neurotransmitters, mitigating oxidative stress, repairing intestinal barrier damage, and increasing tight junction protein expression. This study represents the first use of red pine seed direct-drinking oil to alleviate constipation in mice, providing a novel approach to improving symptoms in individuals with constipation.

## 1. Introduction

Constipation is a globally prevalent condition characterized by infrequent bowel movements (fewer than three times per week), accompanied by dry stools, difficulty in defecation, and other symptoms [1]. With the acceleration of modern lifestyles and changes in dietary habits, the incidence of constipation has been rising [2]. The global average prevalence of constipation is approximately 15%, with rates among older adults ranging from 15% to 50% [3]. Constipation significantly impacts and reduces patients’ health and quality of life. Reports indicate that individuals with constipation have an increased risk of developing Parkinson’s disease, cardiovascular diseases, and colorectal cancer [4,5].

Current treatments for constipation include dietary modifications, medications, biofeedback therapy, and surgical interventions [6]. However, most constipation medications, such as laxatives, can lead to side effects, including diarrhea and excessive bloating. Biofeedback therapy, while potentially effective, has limitations due to significant variations in outcomes, prolonged treatment cycles, and high costs. Surgical treatments for constipation may also pose risks, including postoperative pain and bleeding [6,7]. In recent years, there has been a growing public preference for dietary and nutritional interventions in managing gastrointestinal diseases. Studies suggest that dietary adjustments may offer a promising approach to managing chronic conditions [8,9]. Red pine seed oil, a major component of red pine kernels, contains approximately 90% unsaturated fatty acids, including linolenic acid, linoleic acid, eicosapentaenoic acid, arachidonic acid, and a unique fatty acid—pinolenic acid [10,11]. Rich in bioactive compounds with medicinal properties, red pine seed oil has been reported to have laxative, anti-inflammatory, and constipation-relieving effects, offering the potential for further scientific and commercial development [12,13].

Direct-drinking oil refers to edible oils that can be consumed directly, unlike conventional oils that require cooking with food. As a dietary fat supplement, direct-drinking oil can be consumed independently. It is typically produced from high-quality oilseeds and processed without high-temperature refining, thereby retaining its natural nutrients and unique flavor. This method of consumption addresses the degradation of lipid nutrients caused by prolonged heating during cooking, which reduces the quality of conventional oils [14,15]. Red pine seed oil is rich in polyunsaturated fatty acids but contains relatively low levels of saturated fatty acids, resulting in poor oxidative stability and an unbalanced fatty acid composition. In contrast, coconut oil is high in saturated fatty acids, camellia oil contains a high proportion of monounsaturated fatty acids, and rice bran oil is rich in n − 6 fatty acids. Red pine seed direct-drinking oil is a novel blended edible oil formulated using red pine seed oil as the base oil, combined with coconut oil, rice bran oil, and camellia oil in specific proportions. This formulation aims to create an oil product that meets the fatty acid ratio recommended by the American Heart Association (SFA:MUFA:PUFA = 1:1:1, n − 6:n − 3 = (4–6):1). Additionally, it aligns with the nutritional principles of Mediterranean and ketogenic diets [16,17], offering high oxidative stability, suitability for direct consumption, and economic feasibility. In some regions of China, such as Fujian, camellia oil has been used traditionally to treat constipation [15]. Studies have shown that coconut oil alleviates loperamide-induced constipation in rats [18], and rice bran oil reduces gastrointestinal inflammation and promotes gut health [19]. Dietary therapy has become a research focus, and the balanced fatty acid composition and rich nutrient content of red pine seed direct-drinking oil make it a promising option. However, its role in treating constipation and the underlying mechanisms remain unclear. This study establishes a loperamide-induced constipation mouse model to explore whether red pine seed direct-drinking oil can alleviate constipation, providing a theoretical basis for its use as a dietary therapy and functional food.

## 2. Materials and Methods

### 2.1. Materials and Instruments

BALB/c mice: Male, SPF grade, 7 weeks old, 56 in total, obtained from Changchun Yisi Experimental Animal Technology Co., Ltd., Changchun, China. All animal experimental procedures were approved by the Animal Ethics Committee of Harbin University of Commerce (Approval No. HSDU2020–065, Harbin, China).

Red pine seeds (*Pinus koraiensis* Sieb.et Zucc) were provided by Heilongjiang Qinghe Forestry Bureau Co., Ltd., Harbin, China. Coconut oil, rice bran oil, and camellia oil were purchased from BEAUTY MART in Harbin, China. Loperamide hydrochloride was obtained from Shanghai Yuanye Biotechnology Co., Ltd., Shanghai, China. Mosapride was sourced from Macklin. Assay kits for substance P (SP), endothelin-1 (ET-1), and vasoactive intestinal peptide (VIP) were purchased from Shanghai Enzyme-linked Biotechnology Co., Ltd., Shanghai, China. Kits for nitric oxide (NO), superoxide dismutase (SOD), and catalase (CAT) were acquired from Nanjing Jiancheng Bioengineering Institute, Nanjing, China. RNA extraction reagent, RIPA lysis buffer, and BCA protein quantification assay kits were obtained from Wuhan Servicebio Technology Co., Ltd., Wuhan, China.

Gas chromatography-mass spectrometry (GC-MS) 7890-5973N (Agilent, Santa Clara, CA, USA), a benchtop high-speed refrigerated microcentrifuge (DragonLab, Beijing, China), a quantitative real-time PCR system (Bio-Rad, Hercules, CA, USA), and a PCR instrument (Beijing Dongsheng Innovation Biotechnology Co., Ltd., Beijing, China) were used.

### 2.2. Preparation of Red Pine Seed Direct-Drinking Oil

Preparation of red pine seed oil: Pine seeds were shelled, dried in a 40 °C forced-air oven until a constant weight was reached, and the red seed coat was removed. The treated pine seeds were ground into powder, and a 1:5 g/mL solid-to-solvent ratio of hexane was added. After a 12 h extraction, the solution was filtered, and the filtrate was concentrated under vacuum using rotary evaporation at 45 °C until a constant weight was obtained, yielding the red pine seed oil.

Using laboratory-prepared red pine seed oil, as well as commercially purchased camellia oil, rice bran oil, and coconut oil, a mathematical model was constructed with MATLAB 2022a (MathWorks, Natick, MA, USA) to design the formulation. Oils were precisely weighed according to the designed formula and mixed using a magnetic stirrer at 40 °C with a speed of 800 rpm for 30 min. The model is as follows:

*A_jK_* represents the content of the *j*-th fatty acid in the *K*-th base oil; *n* represents the number of base oils; *A*_1*K*_ represents SFA; *A*_2*K*_ represents MUFA; *A*_3*K*_ represents PUFA; *A*_4*K*_ represents n − 6; *A*_5*K*_ represents n − 3. The total mass of the blended oil was set to 100 g, and *X_K_* is the content of the *K*-th base oil. The conditions were defined as follows:

$$\sum A_{1K}X_{K}:\sum A_{2K}X_{K}:\sum A_{3K}X_{K}=1$$

$$\sum A_{4K}X_{K}:\sum A_{5K}X_{K}=C\;(C\;\mathrm{is}\;a\;\mathrm{constant}\;\mathrm{of}\;4,5,6)$$

$$\sum X_{K}=100\;\left(X_{K}\geq 0,K=1,2,3.\ldots ,n\right)$$

Transforming into a linear model:

$$\left(A_{11}-A_{21}\right)X_{1}+\left(A_{12}-A_{22}\right)X_{2}+\cdots +\left(A_{1n}-A_{2n}\right)X_{n}=0$$

$$\left(A_{11}-A_{31}\right)X_{1}+\left(A_{12}-A_{32}\right)X_{2}+\cdots +\left(A_{1n}-A_{3n}\right)X_{n}=0$$

$$\left(A_{41}-A_{51}\right)X_{1}+\left(A_{42}-A_{52}\right)X_{2}+\cdots +\left(A_{4n}-A_{5n}\right)X_{n}=0$$

$$X_{1}+X_{2}+X_{3}+\cdots +X_{n}=100\;\left(X_{K}\geq 0,K=1,2,3.\ldots ,n\right)$$

### 2.3. Determination of Physicochemical Indicators

The acid value was determined according to AOAC 940.28, and the peroxide value was measured following AOAC 965.33.

### 2.4. Determination of Fatty Acid Composition

Fatty Acid Methylation: Fatty acids were methylated according to the method described by Zhou [20].

Fatty Acid Analysis: A DB-5 MS fused silica capillary column (30 m × 250 μm × 0.25 μm) was used [20]. The column temperature program was set as follows: initial temperature of 60 °C, increased at 10 °C/min to 130 °C, held for 5 min, then increased at 5 °C/min to 180 °C, and finally at 2.5 °C/min to 250 °C, held for 5 min. The injection port temperature was 250 °C, and the gas chromatography-mass spectrometry (GC-MS) interface temperature was 250 °C. Helium was used as the carrier gas with a flow rate of 1.0 mL/min, a split ratio of 20:1, and an injection volume of 1 μL. Mass spectrometry was conducted with an electron impact ionization energy of 70 eV and a mass-to-charge ratio (*m/z*) range of 50–550.

### 2.5. Animal Experiment Design

**Figure 1:**
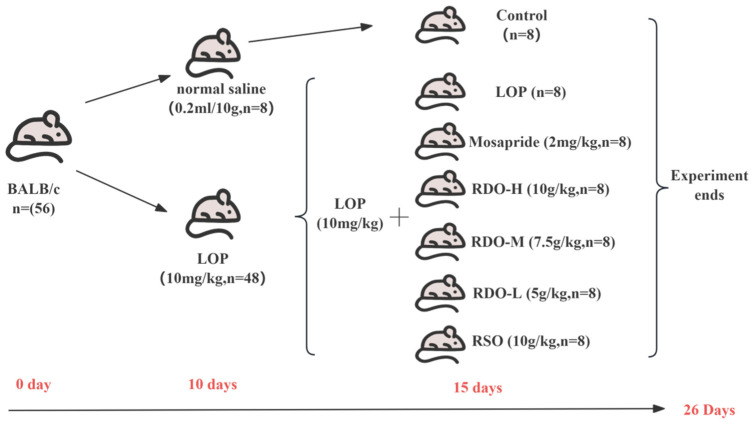
Schematic diagram of the mouse experiment.

As shown in Figure 1, a total of 56 seven-week-old SPF male BALB/c mice were selected and housed in cages at 22 ± 2 °C with a 12 h light–dark cycle, with access to water and standard feed. After one week of acclimatization, the mice were randomly divided into two groups: control (*n* = 8) and loperamide-induced constipation model group (LOP, *n* = 48) (10 mg/kg bw). Constipation was induced using intragastric administration of loperamide hydrochloride once daily for 10 days. The control group received an equivalent volume of saline. Constipation in mice was assessed based on reduced stool output, hard stools, increased defecation time, rough fur, and lethargy. After model establishment, 48 mice were randomly divided into six groups (*n* = 8 per group): LOP group (LOP) (10 mg/kg bw), positive control group (Mosapride) (2 mg/kg bw), red pine seed oil group (RSO) (10 g/kg bw), high-dose group (RDO-H) (10 g/kg bw), medium-dose group (RDO-M) (7.5 g/kg bw), and low-dose group (RDO-L) (5 g/kg bw). To maintain model stability, loperamide was administered during the treatment period with a 4 h interval between loperamide and the test compounds, for a total of 15 days.

### 2.6. Measurement of Defecation Time and Fecal Water Content in Mice

On day 22 of the experiment, the body weight of each mouse was measured at 8:00 AM. The mice were fasted overnight with free access to water. After 18 h, 0.25 mL of activated charcoal suspension was administered via gavage. Each mouse was then placed in a clean, empty cage (one mouse per cage) with free access to food and water. The time interval from the charcoal gavage to the first black stool was recorded. Feces were collected for each mouse over a 2 h period, and the number of fecal pellets was recorded. The feces were then dried at 70 °C for 3 days. Fecal water content was calculated using the following formula [21]:

$$Fecal\;Water\;Content\;\left(\%\right)=\frac{Wet\;Weight-Dry\;Weight}{Wet\;Weight}\times 100\%$$

### 2.7. Gastrointestinal Motility Test in Mice

After the final administration, body weights of each mouse were measured at 8:00 AM, followed by overnight fasting with free access to water. After 18 h, 0.25 mL of activated charcoal suspension was administered via gavage. Mice were euthanized by cervical dislocation 30 min after charcoal gavage. The abdominal cavity was opened along the midline to measure the total length of the intestine (from pylorus to rectum) and the distance traveled by the charcoal [22,23]. The gastrointestinal transit rate (*GI*) was calculated using the formula:

$$GI\;\left(\%\right)=\frac{Charcoal\;Propulsion\;Length}{Total\;Intestinal\;Length}\times 100\%$$

### 2.8. Serum Neurotransmitter Levels in Mice (SP, ET-1, and VIP)

Blood samples were collected from the inferior vena cava and centrifuged (3000 rpm, 15 min) to obtain serum. Commercial ELISA kits were used to add the corresponding reagents following the manufacturer’s instructions. The levels were measured and calculated accordingly.

### 2.9. Serum Levels of NO, SOD, and CAT in Mice

Blood samples were collected from the inferior vena cava and centrifuged (3000 rpm, 15 min) to obtain serum. Commercial kits (Nanjing Jiancheng Bioengineering Institute) were used to measure the levels of superoxide dismutase (SOD), catalase (CAT), and nitric oxide (NO) in the serum.

### 2.10. Histological Analysis of Mice Tissue

Approximately 2 cm of distal colon tissue was fixed in 4% paraformaldehyde buffer for 48 h, embedded in paraffin, and sectioned continuously at a thickness of 4 μm. The paraffin sections were deparaffinized and rehydrated using xylene and ethanol, followed by hematoxylin–eosin (H&E) staining [24]. Pathological changes in colon tissues were observed under a microscope, and images were recorded.

### 2.11. Real-Time PCR for Detection of Occludin and Claudin-1 mRNA Expression in Mice

The expression levels of relevant genes in mouse colon tissue were measured using RT-qPCR. RNA was extracted using the Trizol method, and cDNA was synthesized according to the reverse transcription kit instructions. Primer sequences were synthesized by Wuhan Sevier Biotechnology Co., Ltd. The relative expression levels of occludin and claudin-1 in mouse colon tissue were measured using a real-time quantitative PCR instrument. The primer sequences for occludin, claudin-1, and the reference gene β-actin are shown in Table 1.

### 2.12. Western Blot Analysis of Occludin and Claudin-1 Expression in Mice Colonic Tissue

Protein Extraction: Tissue samples were washed 1–2 times with pre-cooled PBS, cut into small pieces, and placed in a grinding tube with three 3 mm grinding beads. Lysis buffer (10 times the volume of the tissue) was added, and the samples were ground using a homogenizer. The homogenized samples were placed on ice for 30 min, then centrifuged at 12,000 rpm for 10 min at 4 °C. The supernatant was collected as the total protein solution, and protein concentration was determined using a BCA protein assay kit.

Western Blot: An 8% SDS-PAGE gel was prepared, and 10 μL of protein samples were loaded onto the gel. Electrophoresis was conducted at 200 V for 30 min. Proteins were transferred to a PVDF membrane using a wet transfer method at a constant current of 300 mA for 30 min. The PVDF membrane was blocked at room temperature for 30 min, followed by the addition of occludin and claudin-1 primary antibodies and incubation at 4 °C overnight. After 12 h, the membrane was washed with TBST three times, each for 5 min, then incubated with a secondary antibody at room temperature for 30 min before performing ECL detection. Data analysis was conducted using AIWBwell^TM^ analysis software (Servicebio, China).

### 2.13. Statistical Analysis

Statistical analysis was performed using SPSS 26.0 software. Data are expressed as mean ± standard deviation (SD). One-way ANOVA was used for comparisons between groups, and a *p*-value < 0.05 was considered statistically significant.

## 3. Results

### 3.1. Fatty Acid Composition of Red Pine Seed Oil and Its Formulated Oils

As shown in Table 2, the predominant fatty acids differ across the four oils, with a total of 22 fatty acids identified. Red pine seed oil has the highest content of polyunsaturated fatty acids (PUFAs), at 67.40 ± 0.91%. Camellia oil contains a high proportion of monounsaturated fatty acids (MUFAs), reaching 83.83 ± 0.46%. Coconut oil is rich in saturated fatty acids (SFAs), with a content of 94.96 ± 0.64%. In rice bran oil, the proportions of different fatty acids are nearly equal. Notably, except for red pine seed oil, the other three oils contain a significant amount of n − 6 fatty acids, with a content of 30.25 ± 26%.

For red pine seed oil, the ratio of SFAs, MUFAs, and PUFAs is 1:2.77:7.59, and the ratio of n − 6 to n − 3 fatty acids is 2.63:1, which do not align with the fatty acid intake recommended ratios of the Chinese Nutrition Society. Therefore, camellia oil, rice bran oil, and coconut oil were blended with red pine seed oil to achieve a nutritionally rich direct-drinking oil with a balanced fatty acid composition.

### 3.2. Selection of Oil Sample Ratios for Direct-Drinking Oil

Red pine seed oil was used as the base oil and blended with coconut oil, rice bran oil, and camellia oil to achieve the recommended fatty acid ratio of SFA:MUFA:PUFA = 1:1:1, n − 6:n − 3 = (4–6):1, as suggested by the American Heart Association. Using Matlab software 9.12, the final blend proportions were calculated as follows: red pine seed oil (35.95%), camellia oil (15.60%), rice bran oil (26.02%), and coconut oil (22.43%). This study’s direct-drinking oil is formulated from these four different oils to achieve a balanced fatty acid profile.

### 3.3. Validation Experiment

**Figure 2:**
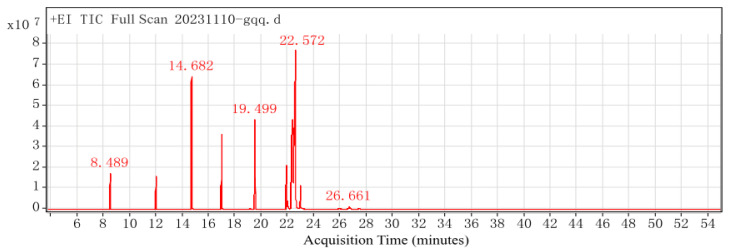
GC-MS chromatogram of red pine seed direct-drinking oil.

The formulated red pine seed direct-drinking oil underwent fatty acid analysis, and the results are shown in Figure 2.

The results, summarized in Table 3, indicate that the measured fatty acid ratios in the direct-drinking oil are SFA:MUFA:PUFA = 1.14:1.08:1, n − 6:n − 3 = 4.17:1. The measured fatty acid ratios closely match the predicted values.

According to the “Dietary Nutrient Reference Intakes for Chinese Residents”, fatty acid intake is calculated as a percentage of total energy intake. To compare the fatty acid content of the direct-drinking oil with the recommended intake, the fatty acid composition of the oil was converted into its percentage of total energy. The recommended intake for fatty acids is 20–30% of total energy, with a median value of 25% used for calculation. The relationship between the percentage of total energy and the content of fatty acid A is expressed as:

$$Energy\;percentage\;of\;A=\left(Fatty\;acid\;content\;of\;A\right)\times 25\%$$

The percentage of total energy provided by the fatty acids in the direct-drinking oil is shown in Table 4.

The energy percentage provided by saturated fatty acids (SFA) in the direct-drinking oil is 8.85%, which is less than the recommended 10%. The energy contribution from n − 6 polyunsaturated fatty acids (PUFAs) is 6.26%, which falls within the recommended range of 2.5–9.0%. Similarly, the n − 3 PUFA energy contribution is 1.50%, meeting the recommended range of 0.5–2.0%. Therefore, the proportions of SFA, n − 6 PUFA, and n − 3 PUFA in this direct-drinking oil are in accordance with the balance requirements outlined in the “Dietary Nutrient Reference Intakes for Chinese Residents”.

### 3.4. Determination of Physicochemical Properties of Red Pine Seed Direct-Drinking Oil and Its Formulated Oils

According to the National Food Safety Standard for Vegetable Oils (GB 2716-2018) [25], the peroxide value for vegetable oils must be below 0.25 g/100 g, and the acid value should be less than 3 mg/g. Based on the data presented in Table 5, the peroxide and acid values of the red pine seed direct-drinking oil and its formulated oils met these standards. The direct-drinking oil shows a reduction in both acid and peroxide values compared to the red pine seed oil, with a 46.15% decrease in the peroxide value. Therefore, the direct-drinking oil exhibits improved oxidative stability compared to red pine seed oil.

### 3.5. Effects of Red Pine Seed Direct-Drinking Oil on Baseline Indicators of Constipated Mice

#### 3.5.1. Effects of Red Pine Seed Direct-Drinking Oil on the Body Weight of Constipated Mice

**Figure 3:**
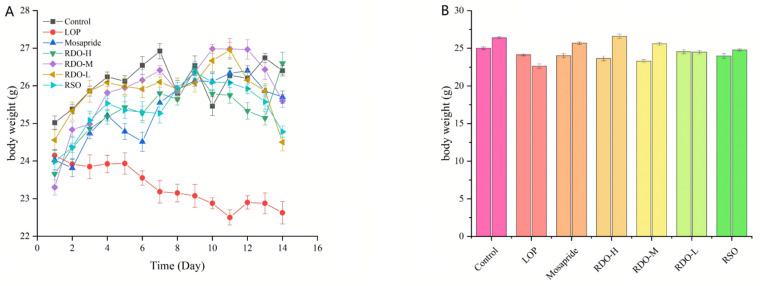
Changes in mouse body weight (**A**,**B**) (Control: control group; LOP: model group; Mosapride: positive control group; RDO-H: high-dose red pine seed direct-drinking oil group; RDO-M: medium-dose red pine seed direct-drinking oil group; RDO-L: low-dose red pine seed direct-drinking oil group; RSO: red pine seed oil group).

As shown in Figure 3, the body weight of mice in the LOP group decreased continuously over time compared to the control group. This suggests that constipation leads to difficulty in defecation, which in turn causes weight loss in mice. In comparison to the LOP group, the body weight of mice in both the direct-drinking oil and red pine seed oil groups increased, with the RDO-H group experiencing the greatest weight gain. While red pine seed oil improved the body weight of constipated mice, the overall effect was not as pronounced as that of the direct-drinking oil group. In conclusion, the red pine seed direct-drinking oil effectively alleviated weight loss caused by constipation in mice.

#### 3.5.2. Effects on Defecation Frequency, Fecal Water Content, and Time to First Dark Stool in Mice

**Figure 4:**
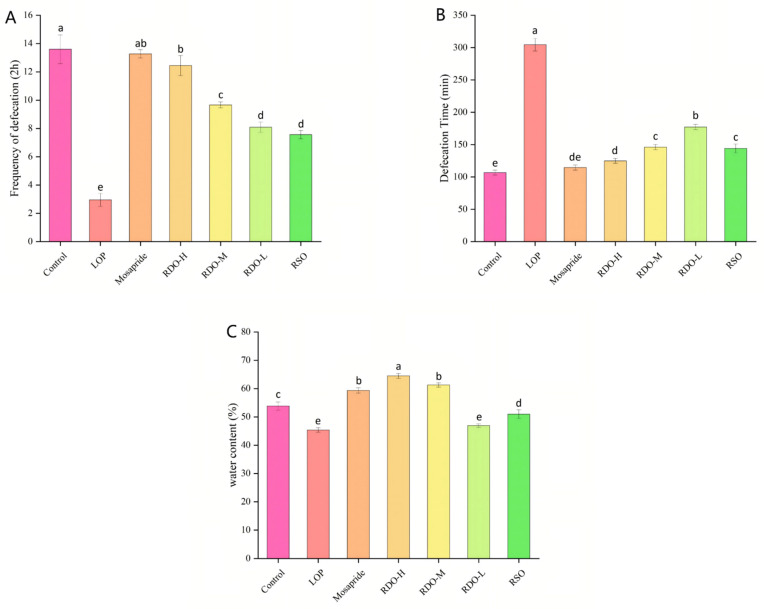
Defecation frequency (**A**), time to first dark stool (**B**), and fecal water content (**C**) in mice. Values with different letters indicate significant differences (*p* < 0.05).

As shown in Figure 4, compared to the LOP group, the control group exhibited a 78.31% reduction in defecation frequency over 2 h (*p* < 0.05), a 15.83% decrease in fecal water content (*p* < 0.05), and a significant 185.65% delay in the time to the first dark stool (*p* < 0.05). After intervention with the direct-drinking oil, the time to first dark stool was significantly shortened, and defecation frequency was increased (*p* < 0.05). Compared to the LOP group, the fecal water content in the RDO groups (RDO-H, RDO-M, and RDO-L) significantly increased to 64.5 ± 0.86%, 61.28 ± 0.74%, and 46.93 ± 0.74%, respectively (*p* < 0.05). In particular, the RDO-H and RDO-M groups showed even better results than the control group (53.86 ± 1.44%) (*p* < 0.05). In summary, red pine seed direct-drinking oil alleviates constipation in mice by shortening defecation time, increasing defecation frequency, and enhancing fecal water content. Furthermore, the higher the dose of direct-drinking oil, the more significant the relief of constipation. The results indicate that while red pine seed oil also alleviates constipation, its overall efficacy is inferior to the higher and medium doses of red pine seed direct-drinking oil.

#### 3.5.3. Effects on Intestinal Propulsion Rate in Mice

**Figure 5:**
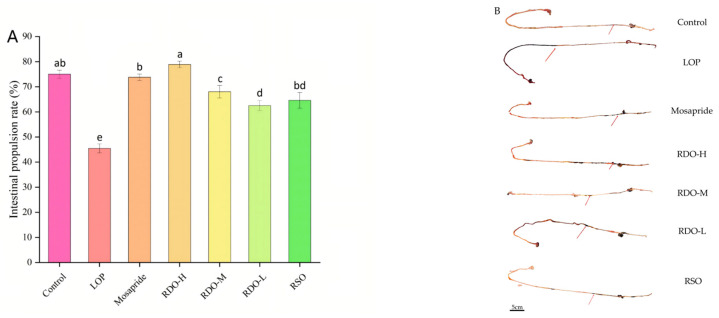
Intestinal propulsion rate (**A**) and intestinal propulsion length (**B**), with the red arrow indicating the location of ink propulsion. Values with different letters indicate significant differences (*p* < 0.05).

As shown in Figure 5, the intestinal propulsion rate in the control group was 75.02 ± 1.60%. In contrast, the LOP group exhibited a significant decrease of 39.40% (*p* < 0.05), with an intestinal propulsion rate of 45.46 ± 1.78%. Compared to the LOP group, the intestinal propulsion rate in the RKO-H group significantly increased by 73.54% (*p* < 0.05), reaching 78.89 ± 1.28%. Among all six groups, the RDO-H group had the highest intestinal propulsion rate. These findings indicate that different doses of red pine seed direct-drinking oil significantly alleviate constipation in mice, with higher doses leading to more pronounced improvements.

### 3.6. Effects of Red Pine Seed Direct-Drinking Oil on Neurotransmitters

**Figure 6:**
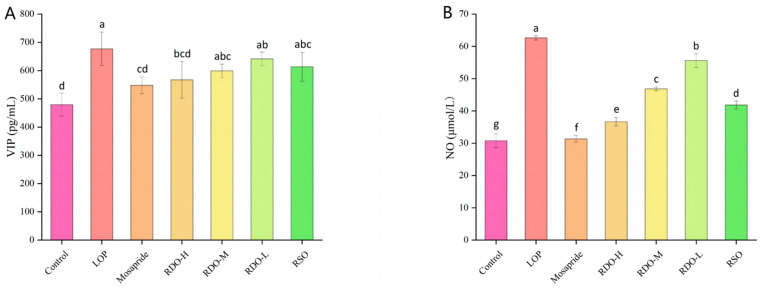

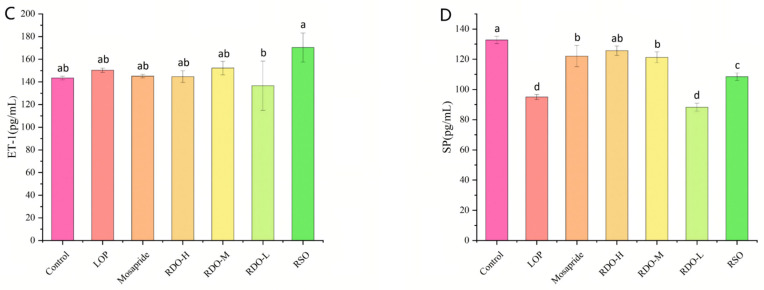
Serum levels of vasoactive intestinal peptide (VIP), nitric oxide (NO), endothelin-1 (ET-1), and substance P (SP) (**A**–**D**). Values with different letters indicate significant differences (*p* < 0.05).

As shown in Figure 6, mice in the LOP group exhibited significantly higher serum levels of vasoactive intestinal peptide (VIP) and NO (*p* < 0.05), as well as endothelin-1 (ET-1), compared to the control group. However, SP levels were significantly lower (*p* < 0.05). After intervention with RDO, these changes were reversed. Compared to the LOP group, the RDO-H group showed the most pronounced improvements, with VIP and NO levels decreasing by 109.72 pg/mL (*p* < 0.05) and 25.98 μmol/L (*p* < 0.05), respectively, while SP levels increased by 30.64 pg/mL (*p* < 0.05). However, the effect on ET-1 levels was minimal. Following RDO-L intervention, ET-1 expression significantly decreased (*p* < 0.05). Overall, the RDO-H group achieved the best results in improving constipation in mice.

### 3.7. Effects of Red Pine Seed Direct-Drinking Oil on Oxidative Stress Markers

**Figure 7:**
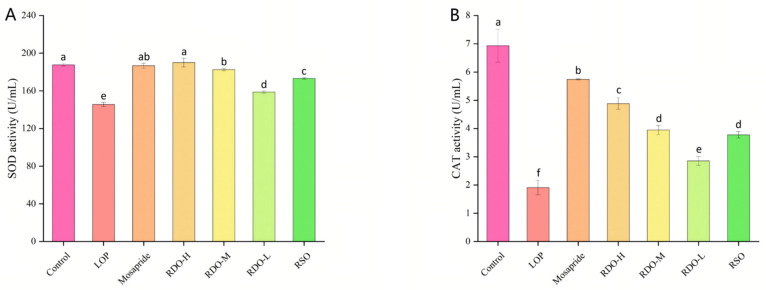
Serum SOD and CAT levels (**A**,**B**). Values with different letters indicate significant differences (*p* < 0.05).

The activities of SOD and CAT in mouse serum were further measured. As shown in Figure 7, the activities of SOD and CAT in the LOP group were significantly lower than those in the control group (*p* < 0.05). After intervention with RDO, SOD activity increased, and the effect became more pronounced with higher doses. In the RDO-H, RDO-M, and RDO-L groups, serum SOD levels increased to 190.05 ± 4.70, 182.57 ± 1.43, and 158.75 ± 1.23 U/mL, respectively (*p* < 0.05), with the RDO-H group exceeding the control group (187.77 ± 1.33 U/mL). Additionally, compared to the LOP group, CAT levels were significantly increased in the RDO-H, RDO-M, and RDO-L groups (*p* < 0.05), with the effect becoming more significant as the dose increased. In summary, RDO can enhance the antioxidant capacity in constipated mice, and its effects are dose-dependent.

### 3.8. Effects of Red Pine Seed Direct-Drinking Oil on Colon Tissue in Constipated Mice

**Figure 8:**
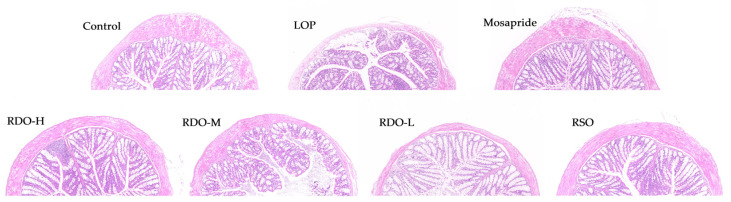
Histological sections of mouse colon tissue (×200).

To investigate the protective effects of red pine seed direct-drinking oil on the intestines, histopathological analysis of colon tissue was conducted, as shown in Figure 8. In the control group, the cells were neatly arranged, the tissue structure was clear and intact, there was no inflammatory cell infiltration in the mucosa, and the muscularis tissue was well-preserved. In contrast, the LOP group exhibited goblet cell damage, reduced goblet cell numbers, inflammatory cell infiltration, severe deformation or disappearance of crypt structures, and reduced mucosal and muscular layer thickness. After intervention with RDO and RSO, the condition of goblet cells improved and returned to normal, crypt structures were restored, inflammatory cell infiltration was inhibited, and the thickness of the mucosal and muscular layers increased significantly. Histopathological observations of colon tissue indicate that RDO can improve colon injury and maintain the integrity of colon tissue in constipated mice.

### 3.9. Effects of Red Pine Seed Direct-Drinking Oil on Occludin and Claudin-1 mRNA and Protein Expression in the Colons of Constipated Mice

**Figure 9:**
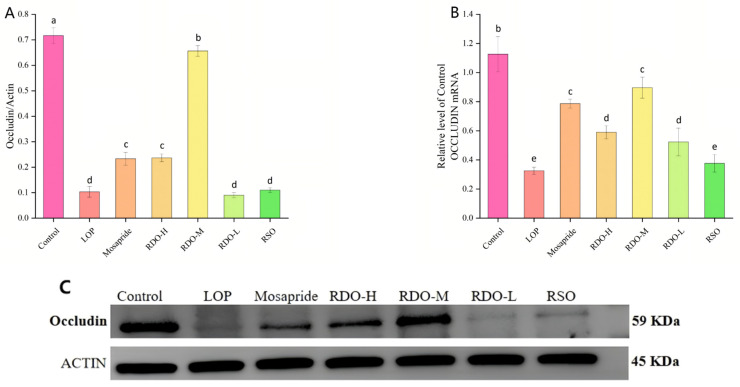
Occludin protein expression levels (**A**), occludin mRNA expression levels (**B**), and western blot analysis of occludin protein expression (**C**). Values with different letters indicate significant differences (*p* < 0.05).

To further explore the effects of red pine seed direct-drinking oil on the intestinal barrier of constipated mice, this study measured the protein and mRNA expression levels of the tight junction proteins occludin and claudin-1 in the mouse colon. As shown in Figure 9, the protein and mRNA expression levels of occludin in the LOP group decreased by 86.11% and 70.80%, respectively, compared to the control group (*p* < 0.05). After intervention with red pine seed direct-drinking oil, the expression levels of occludin protein and mRNA in constipated mice were significantly upregulated. In the RDO-H and RDO-M groups, occludin protein expression and mRNA levels were significantly increased from 0.10 ± 0.02 and 0.33 ± 0.03 in the LOP group to 0.24 ± 0.02 and 0.59 ± 0.05 (*p* < 0.05) and 0.66 ± 0.02 and 0.90 ± 0.07 (*p* < 0.05), respectively. However, the therapeutic effect of RSO was not ideal. In summary, both the high-dose (RDO-H) and medium-dose (RDO-M) interventions of red pine seed direct-drinking oil effectively restored occludin expression, with the RDO-M group showing the best results, almost reaching normal levels.

**Figure 10:**
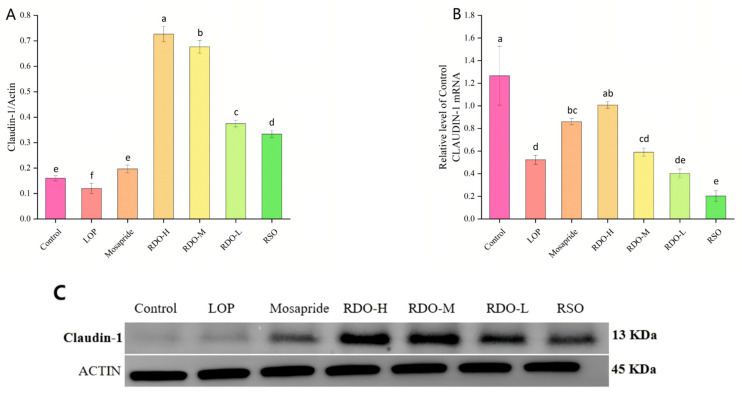
Claudin-1 protein expression levels (**A**), claudin-1 mRNA expression levels (**B**), and western blot analysis of claudin-1 protein expression (**C**). Values with different letters indicate significant differences (*p* < 0.05).

As shown in Figure 10, the protein and mRNA expression levels of claudin-1 in the LOP group were reduced by 25.00% and 59.00%, respectively, compared to the control group (*p* < 0.05). After intervention with red pine seed direct-drinking oil, claudin-1 protein expression and mRNA levels were significantly upregulated. Compared to the control group, the protein expression levels in the RDO-H, RDO-M, and RDO-L groups increased by 0.56, 0.52, and 0.22, respectively (*p* < 0.05), with protein levels significantly exceeding those in the control group (*p* < 0.05). Similarly, mRNA levels in the RDO-H, RDO-M, and RDO-L groups were significantly higher than those in the LOP group, with a dose-dependent effect, while the therapeutic effect of RSO was not satisfactory. In this study, both high and medium doses of red pine seed direct-drinking oil significantly increased claudin-1 protein expression, far exceeding the levels in the control group, with the high-dose intervention showing the best therapeutic effect. In conclusion, RDO enhanced the expression of tight junction proteins in intestinal tissues, improved the tight junctions between intestinal epithelial cells, and alleviated inflammation and intestinal barrier damage caused by constipation by upregulating the expression of occludin and claudin-1 proteins. This may be one of the mechanisms by which red pine seed direct-drinking oil improves intestinal barrier damage and relieves constipation.

## 4. Discussion

In this study, mice with constipation induced by loperamide exhibited clear symptoms, including weight loss, significantly reduced defecation frequency, prolonged defecation time, and decreased fecal water content. Constipation is closely associated with intestinal motility, making the intestinal propulsion rate a critical indicator for assessing intestinal function and determining the severity of constipation in mice [26]. One of the main functions of the intestine is to facilitate the movement of intestinal contents, with the degree of peristalsis determined by the speed of intestinal propulsion [27]. The time to first dark stool, which reflects the total gastrointestinal transit time, indicates the duration of fecal passage through the intestine. Constipation slows intestinal peristalsis, reducing fecal transit speed and causing fecal water to be absorbed, making the stool hard and dry. A shorter time to first dark stool indicates faster bowel movements, suggesting an improvement in constipation [28,29]. Thus, defecation time, frequency, fecal water content, and intestinal propulsion rate are all important indicators of constipation severity. The study results show that red pine seed direct-drinking oil significantly shortens the time to first dark stool and alleviates constipation in mice by increasing the intestinal propulsion rate, defecation frequency, and fecal water content. Moreover, higher doses of red pine seed direct-drinking oil produce more pronounced effects in relieving constipation. Although red pine seed oil also alleviated constipation, its effects were inferior to those of the medium- and high-dose groups of red pine seed direct-drinking oil.

Patients with constipation typically show abnormal neurotransmitter levels [30]. VIP and NO are inhibitory neurotransmitters, and their elevated levels are closely related to constipation [24,31]. VIP relaxes smooth muscle and reduces gastrointestinal motility, contributing to constipation [32]. NO binds to soluble guanylate cyclase, forming an inhibitory junction potential that reduces smooth muscle contraction in the gastrointestinal tract [33]. ET-1 maintains normal intestinal function by regulating vascular tone and cardiovascular stability [34]. Increased levels of excitatory neurotransmitters can enhance intestinal motility and relieve constipation. SP, an excitatory neurotransmitter, stimulates interstitial cells of Cajal, promoting smooth muscle contraction in the gastrointestinal tract. Thus, elevated SP levels increase gastrointestinal contractions and motility [35,36]. Excessive production of inhibitory neurotransmitters and insufficient production of excitatory neurotransmitters inhibit colonic motility, leading to constipation. The balance between inhibitory and excitatory neurotransmitters is crucial for intestinal motility [37]. The results of this study show that, compared to the constipation group, mice treated with red pine seed direct-drinking oil exhibited increased serum SP levels, while VIP, NO, and ET-1 levels decreased, approaching those of the normal group. The RDO-H group demonstrated a greater improvement in constipation symptoms in mice compared to the red pine seed oil (RSO) group. Red pine seed direct-drinking oil may help restore the balance between inhibitory and excitatory neurotransmitters, thereby relieving constipation.

Studies have shown that SOD activity in constipated patients is lower than in the general population, likely due to inflammation in the intestines caused by constipation. Inflammatory conditions in the intestine reduce SOD activity [38]. SOD converts harmful superoxide radicals into hydrogen peroxide [39]. Although hydrogen peroxide is a reactive oxygen species, CAT breaks it down into water, which is harmless to the body. Together, SOD and CAT form an antioxidant chain that mitigates intestinal damage caused by constipation [39]. The intervention of red pine seed direct-drinking oil significantly restored SOD and CAT levels in the serum of constipated mice, with greater effects observed at higher doses. Compared to the red pine seed oil (RSO) group, the RDO-H and RDO-M groups demonstrated superior efficacy in alleviating oxidative stress in constipated mice. These findings suggest that red pine seed direct-drinking oil can alleviate oxidative stress in constipated mice. The results of this study are consistent with those of Yi et al., who reported that fermented soybean milk alleviated loperamide-induced constipation in mice by enhancing antioxidant enzyme activity [34].

The relief of constipation is closely related to the health of the intestinal barrier [40]. Numerous studies have shown that constipation is often accompanied by intestinal inflammation [41]. Constipation is closely related to the thickness of the colon’s muscular layer, with thinning of this layer indicating weakened colon contractility, leading to slowed colonic transit, intestinal dysfunction, and difficulty in defecation, which in turn induces constipation [42,43]. Mucins, synthesized by goblet cells, form a protective mucus layer on the surface of intestinal epithelial cells. The colonic mucosa, composed of dense and organized epithelial cells, plays a crucial role in regulating water absorption to maintain water and electrolyte balance in the intestine. The mucosal barrier protects the gastrointestinal tract by preventing increased permeability due to inflammation [44,45]. Histological analysis shows that red pine seed direct-drinking oil treatment reduced inflammatory infiltration, significantly increased the thickness of the muscular layer, restored the number of goblet cells, and helped the colon tissue return to normal in constipated mice. These findings suggest that red pine seed direct-drinking oil can repair colon damage caused by constipation. Thus, maintaining the integrity of the intestinal barrier may be a potential mechanism through which red pine seed direct-drinking oil alleviates constipation. This study aligns with the results of Shi et al., which demonstrated that a compound beverage alleviates colonic damage to relieve constipation in mice [2].

To further investigate the role of red pine seed direct-drinking oil in the mechanical barrier of the intestines in constipated mice, this study measured the protein and mRNA expression levels of the tight junction proteins occludin and claudin-1 in the mouse colon. The intestine is the largest internal organ in contact with the external environment. The complex structure of the intestinal barrier consists of four components: the microbial barrier, physical barrier, chemical barrier, and immune barrier [45]. The physical barrier, formed by intestinal epithelial cells and their tight junctions (TJs), prevents bacteria and macromolecules from crossing the intestinal wall into the body [46]. TJs, which include proteins such as occludin and claudin, are critical for maintaining the integrity of the intestinal barrier [47]. Additionally, the structure of TJs is closely related to intestinal inflammation. Studies have shown that the disruption of TJ integrity leads to the activation of immune cells and the release of inflammatory factors [48]. The results of this study demonstrated that although red pine seed oil increased the expression of occludin and claudin-1, red pine seed direct-drinking oil led to a greater upregulation of tight junction protein expression in the colons of constipated mice. By maintaining the structural and functional integrity of epithelial cells, red pine seed direct-drinking oil repaired the physical barrier of the intestine and alleviated inflammation and intestinal barrier damage caused by constipation through the regulation of the occludin/claudin-1 pathway. This mechanism may underlie the ability of red pine seed direct-drinking oil to alleviate constipation symptoms.

## 5. Conclusions

A mathematical model was developed to estimate the percentage composition of four oils: red pine seed oil (35.95%), camellia oil (15.60%), rice bran oil (26.02%), and coconut oil (22.43%). Subsequent experimental validation showed that the predicted values of the direct-drinking oil closely matched the measured values. Animal experiments demonstrated that the medium- and high-dose groups of the laboratory-prepared red pine seed direct-drinking oil improved constipation symptoms in mice, outperforming pure red pine seed oil. These effects included increased fecal water content, enhanced defecation frequency, higher intestinal transit rate, and reduced first defecation time of black stool (*p* < 0.05). The direct-drinking oil also reduced serum levels of the inhibitory neurotransmitters VIP, NO, and ET-1 in constipated mice, while significantly increasing the excitatory neurotransmitter SP in the medium- and high-dose groups (*p* < 0.05), thereby synergistically regulating intestinal motility. In addition, the red pine seed direct-drinking oil significantly increased the serum levels of the antioxidant enzymes SOD and CAT (*p* < 0.05), enhancing the antioxidant capacity of constipated mice. This effect was more pronounced with higher doses. Histological examination (HE staining) indicated that red pine seed direct-drinking oil effectively repaired colonic damage and alleviated intestinal inflammation in constipated mice. Furthermore, it significantly upregulated the expression of occludin and claudin proteins and their corresponding mRNAs (*p* < 0.05), reinforcing the intestinal mechanical barrier.

These findings indicate that red pine seed direct-drinking oil improves constipation symptoms in mice, potentially through mechanisms involving enhanced gastrointestinal motility, neurotransmitter modulation, increased antioxidant capacity, intestinal barrier repair, and inflammation reduction.

## Figures and Tables

**Table 1:** Primer sequences.

Target Genes	Primer Sequences (5′-3′)	Accession Number
*Claudin-1*	F:GTGTCCTACTTTCCTGCTCCTGT R:TCACACATAGTCTTTCCCACTAGAAG	NM_016674.4
*Occludin*	F:TCTGACTATGCGGAAAGAGTTGA R:CTCTAGGTTACCATTGCTGCTGT	NM_001360536.1
*β-actin*	F:GTGACGTTGACATCCGTAAAGA R:GTAACAGTCCGCCTAGAAGCAC	NM_007393.3

**Table 2:** Fatty acid composition and mass fraction (%) of the formulated oils.

Fatty Acid	Red Pine Seed Oil	Camellia Oil	Rice Bran Oil	Coconut Oil
Caprylic acid (C_8:0_)	-	-	-	9.10 ± 0.26
Capric acid (C_10:0_)	-	-	-	7.68 ± 0.33
C_11:0_	-	-	-	0.03 ± 0.01
C_11:1_	-	-	0.01 ± 0.00	-
Lauric acid (C_12:0_)	-	-	-	47.34 ± 0.56
C_13:0_	-	-	-	0.04 ± 0.01
C_14:0_	0.03 ± 0.01	0.03 ± 0.01	0.42 ± 0.09	19.16 ± 0.16
C_15:0_	0.01 ± 0.00	-	0.04 ± 0.01	-
C_16:1_	0.09 ± 0.01	0.07 ± 0.01	0.23 ± 0.06	-
C_16:2_	-	-	0.01 ± 0.00	-
Palmitic acid (C_16:0_)	5.96 ± 0.15	4.42 ± 0.33	25.00 ± 0.15	8.65 ± 0.22
C_17:0_	0.01 ± 0.00	-		-
C_17:1_	-	-	0.02 ± 0.01	-
Stearic acid (C_18:0_)	2.76 ± 0.58	3.15 ± 0.21	2.13 ± 0.66	2.98 ± 0.15
Oleic acid (C_18:1_)	23.28 ± 0.05	83.56 ± 0.37	39.73 ± 0.59	4.40 ± 0.16
Linoleic acid (C_18:2_)	47.30 ± 0.65	6.84 ± 0.25	30.25 ± 0.26	0.64 ± 0.07
Linolenic acid (C_18:3_)	17.94 ± 0.42 (Pinolenic acid)	0.46 ± 0.10	-	-
C_19:0_	-	-	0.01 ± 0.00	-
Arachidic acid (C_20:0_)	0.11 ± 0.06	0.02 ± 0.01	0.86 ± 0.05	-
C_20:1_	1.21 ± 0.02	0.19 ± 0.01	0.33 ± 0.06	-
C_20:2_	0.26 ± 0.03	-	-	-
C_20:3_	1.28 ± 0.01	-	-	-
C_21:0_	-	-	-	-
C_21:1_	-	-	-	-
C_22:0_	-	0.85 ± 0.03	0.25 ± 0.01	-
C_22:1_	-	0.01 ± 0.00		-
C_22:2_	0.62 ± 0.05	-		-
C_24:0_	-	0.18 ± 0.01	0.29 ± 0.03	-
SFA	8.88 ± 0.23	8.65 ± 0.36	28.99 ± 0.08	94.96 ± 0.64
MUFA	24.58 ± 0.06	83.83 ± 0.46	40.31 ± 0.37	4.40 ± 0.07
PUFA	67.40 ± 0.91	7.30 ± 0.41	30.26 ± 0.31	0.64 ± 0.07
n − 6	47.30 ± 0.65	6.84 ± 0.25	30.25 ± 26	0.64 ± 0.07
n − 3	17.94 ± 0.42	0.46 ± 0.10	-	-

“-”, indicates not detected.

**Table 3:** Verification of fatty acid composition using mathematical model.

Value	Mass Fraction (%)				
	SFA	MUFA	PUFA	n − 6	n − 3
Predicted	33.33	33.33	33.33	26.67	6.67
Measured	35.40	33.54	31.06	25.05	6.01

**Table 4:** Fatty acid energy percentage in direct-drinking oil.

Item	Direct-Drinking Oil (%)	Recommended Value (%)	Recommended Range (%)
SFA Energy Percentage	8.85	/	<10
MUFA Energy Percentage	8.34	10.00	/
PUFA Energy Percentage	7.77	10.00	/
n − 6 PUFA Energy Percentage	6.26	4.00	2.5–9.0
n − 3 PUFA Energy Percentage	1.50	0.60	0.5–2.0

**Table 5:** Acid value and peroxide value of the sample oils.

Sample	Acid Value (mg/g)	Peroxide Value (g/100 g)
Direct-Drinking Oil	0.30 ± 0.05	0.07 ± 0.01
Red Pine Seed Oil	0.31 ± 0.06	0.13 ± 0.02
Camellia Oil	0.15 ± 0.02	0.11 ± 0.01
Rice Bran Oil	0.20 ± 0.04	0.07 ± 0.02
Coconut Oil	0.44 ± 0.01	0.03 ± 0.01

## Data Availability

The original contributions presented in the study are included in the article, further inquiries can be directed to the corresponding authors.
